# Comparison of microwave ablation and parathyroidectomy for treating severe secondary hyperparathyroidism

**DOI:** 10.3389/fendo.2025.1424248

**Published:** 2025-03-10

**Authors:** Shuiping Li, Jincheng Qiu, Xiaoguang Zhang, Fuzhen Wang, Xianrong Yang, Xiaoyan Chen, Xiaofang Guo, Zuolin Li, Min Lin, Xiaolian Li, Jinghua He, Guorong Lyu, Jiantang Zhang

**Affiliations:** ^1^ Department of Ultrasound, Longyan First Affiliated Hospital of Fujian Medical University, Longyan, China; ^2^ Department of Interventional Ultrasound, The Fifth Clinical Medical College of Henan University of Traditional Chinese Medicine, Zhengzhou People’s Hospital, Zhengzhou, China; ^3^ Department of Nephrology, Longyan First Affiliated Hospital of Fujian Medical University, Longyan, China; ^4^ Department of Thyroid and Breast Surgery, Longyan First Affiliated Hospital of Fujian Medical University, Longyan, China; ^5^ Department of Nuclear Medicine, Longyan First Affiliated Hospital of Fujian Medical University, Longyan, China; ^6^ Department of Ophthalmology, Longyan First Affiliated Hospital of Fujian Medical University, Longyan, China; ^7^ Department of Ultrasound, The Second Affiliated Hospital of Fujian Medical University, Quanzhou, China

**Keywords:** microwave ablation, parathyroidectomy, secondary hyperparathyroidism, bone metabolic markers, bone mineral density

## Abstract

**Objective:**

This study compared the efficacy of microwave ablation (MWA) and parathyroidectomy (PTX) in the treatment of secondary hyperparathyroidism (SHPT) and evaluated the improvement of bone metabolic markers (BMMs) and bone mineral density (BMD).

**Materials and methods:**

Eligible patients with SHPT treated between January 2019 and August 2022 were enrolled in the study and were divided into two groups: MWA and PTX. Outcome measures included the treatment success rate, percentage of patients whose intact parathyroid hormone (iPTH) concentration was within the target range, serum calcium (Ca), phosphorus (P), alkaline phosphatase (ALP), osteocalcin (OC), C-terminal cross-linked telopeptide of type I collagen (β-CXT), and BMD. Data on the procedure time, intraoperative blood loss volume, length and cost of hospitalization, incidence of postoperative complications, and recurrence rates were analyzed.

**Results:**

A total of 107 patients with SHPT—48 in the MWA group and 59 in the PTX group— were included in the study. There were no significant differences in baseline data between the two groups (p>0.05). At the final follow-up, both therapies decreased iPTH, Ca, P, ALP, OC, and β-CXT levels and increased BMD (p<0.05). Nonetheless, the decrease in iPTH, ALP, OC, and β-CXT was more pronounced 6 and 12 months after PTX (p<0.05). The percentage of patients whose iPTH level was within the target range was significantly higher in the MWA group (p<0.05). The incidence of severe hypocalcemia was significantly lower in the MWA group (p<0.05).

**Conclusion:**

MWA can improve BMMs and BMD, and is a minimally invasive approach with great potential for treating patients with SHPT who cannot tolerate PTX.

## 1 Introduction

Secondary hyperparathyroidism (SHPT) is a common complication of chronic renal insufficiency. Patients with SHPT generally present with elevated serum intact parathyroid hormone (iPTH) levels and disturbances in serum calcium (Ca) and phosphorus (P) metabolism. Nonetheless, serum calcium levels are usually normal or sometimes low in secondary hyperparathyroidism, distinguishing SHPT from primary and tertiary hyperparathyroidism (1, 2). These dysfunctions lead to increased bone turnover, decreased bone mineral density (BMD), renal osteodystrophy, and vascular calcification, increasing the risk of fractures and cardiovascular mortality (3, 4).

Early-stage SHPT can be treated with oral active vitamin D, intravenous vitamin D analogs, calcimimetics, dialysis (5–7). Parathyroidectomy (PTX) is effective in patients with SHPT who do not respond to drug treatments (8–11). However, some patients with SHPT do not tolerate general anesthesia because of severe organ dysfunction, and a minimally invasive percutaneous approach is required in these cases. The efficacy of thermal ablation and PTX for treating SHPT is similar (12–15). Nonetheless, the former is performed under local anesthesia, with short operation time, high safety, and faster postoperative recovery (16–18). Moreover, thermal ablation is associated with a lower incidence of permanent parathyroid hypothyroidism and hypocalcemia compared with PTX (12–14).

Bone metabolic markers (BMMs), including biochemical markers, bone metabolism regulators, and bone turnover markers (BTMs), can assess bone metabolic status non-invasively (19, 20). BMD reflects the status of bone tissue and predicts the risk of fractures (21). PTX can improve BMMs and BMD in patients with SHPT (22–25). Nonetheless, the effects of thermal ablation on bone metabolism in these patients are limited to iPTH and Ca and P levels, and no systematic study has investigated changes in BMMs and BMD following thermal ablation. This study compared the effects of PTX and microwave ablation (MWA) on BMMs and BMD in patients with SHPT.

## 2 Materials and methods

### 2.1 Patients

Patients with SHPT admitted from two centers between January 2019 and August 2022 were included in the study and were divided into two groups—PTX and MWA—based on patient preferences. All patients received regular dialysis (dialysis modes included hemodialysis and peritoneal dialysis), and the dialysate calcium concentration was 1.25–1.75 mmol/L. After treatment, all patients received oral calcium carbonate 1.8 g/d, calcitriol 2.0 µg/d, and 2 mg/kg/h calcium gluconate intravenous injection, and calcium dosage was adjusted according to serum calcium concentration. All patients underwent parathyroid ultrasound and ^99m^Tc-sestamibi imaging. This retrospective study was approved by the ethical review committees of both institutions ([2021] Ethics Committee Approval for Scientific Research No. 016 and 2021001020).

The inclusion criteria were (a) patients with severe SHPT (SHPT not controlled by dialysis and medications), (b) age 18–85 years, (c) iPTH >600 pg/mL, and (d) ultrasonography revealing one or more parathyroid hyperplasia. The exclusion criteria were (a) patients who had previously undergone single excision or thermal ablation of parathyroid adenomas; (b) patients with obesity (body mass index ≥25 kg/m^2^), diabetes, or long-term use of glucocorticoids and (c) patients with ectopic parathyroid gland on ^99m^Tc-sestamibi imaging.

### 2.2 MWA

**Figure 1:**
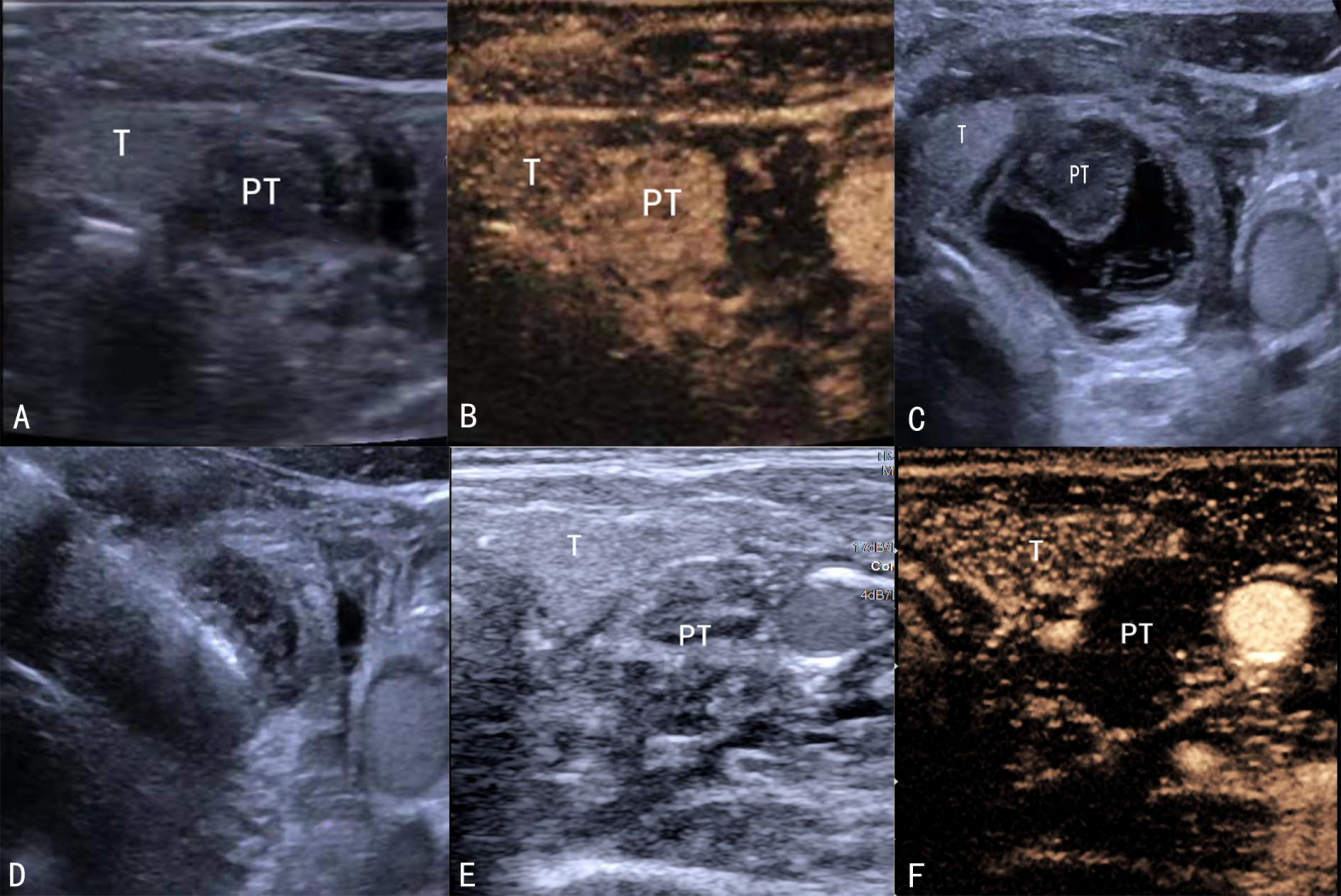
Ultrasound-guided MWA. **(A)** B-scan before ablation showing a hypoechoic parathyroid nodule. **(B)** CEUS showing enhanced parathyroid gland nodules. **(C)** Hydrodissection procedure. **(D)** The probe is inserted in the parathyroid nodule. **(E)** B-scan showing the ablation zone. **(F)** CEUS showing a non-enhancement zone covering the parathyroid nodule. MWA, microwave ablation; CEUS, contrast-enhanced ultrasound.

Ultrasound-guided MWA of parathyroid nodules was performed at each center by an experienced ultrasound interventionist using an ultrasound scanner (Acuson Sequoia; Siemens Healthineers, Erlangen, Germany) and a 4–10 MHz high-frequency linear probe. Contrast-enhanced ultrasound (CEUS) with SonoVue (Bracco, Milan, Italy) was performed before ablation to determine parathyroid blood supply (**Figures 1A, B**). The patient was placed in a supine position, and a 22-G puncture needle was used to inject saline around the parathyroid nodules for hydrodissection until the nodules were separated from the surrounding tissue by >5 mm (**Figure 1C**). A 2.0-mm probe with a 5.0-mm tip (KY-2000; Nanjing Kangyou, Nanjing, China) was inserted in each nodule. Ablation power varied between 25 W and 30 W depending on nodule size. Ablation was performed until the nodules were completely hyperechoic on ultrasonography (**Figure 1D**). Heart rate and blood pressure were closely monitored during ablation. Ablation was stopped immediately if hoarseness or dysphonia occurred. Hematoma and hypoxia were also monitored, and CEUS was performed following ablation of all target nodules (**Figures 1E, F**). Additional ablation was performed immediately in lesions with residual contrast enhancement.

### 2.3 Surgical procedure

PTX includes subtotal PTX (sPTX), total PTX combined with autotransplantation (tPTX+AT), and total PTX (tPTX). All our patients underwent tPTX+AT, which was performed by the same experienced surgeon. After general anesthesia, the patient was placed in a supine position. The skin was incised, the subcutaneous tissue and platysma muscle were incised layer by layer, the flap was separated along the inferior platysma fascia, and the white line was cut at the median cervical line. The thyroid was freed, and all parathyroid glands were removed. Approximately 30–60 mg of parathyroid tissue was removed for use. After sufficient hemostasis, the incision was sutured layer by layer. Parathyroid AT was performed to cut three to four fragments of approximately 1 mm^3^ from the spare parathyroid tissue and was implanted into the muscle layer of the right forearm. The implant sites were marked with silk or metal materials.

### 2.4 Data collection and follow-up

iPTH, Ca, P, and alkaline phosphatase (ALP) levels were measured at baseline, 1 and 7 days after treatment, and 1, 3, 6, and 12 months after treatment. Osteocalcin (OC), 25-hydroxy vitamin D [25(OH)D], beta C-terminal cross-linked telopeptide of type I collagen (β-CTX), and BMD of the lumbar spine (LS), femoral neck (FN), and total hip (TH) were measured before treatment and 12 months after treatment. Data on total hospital stay, hospitalization costs, incidence of complications (hypocalcemia, hoarseness, infection/fever, and hematoma), and recurrence rates were collected and analyzed.

The Kidney Disease Improving Global Outcomes (KDIGO) recommends that iPTH concentrations in patients with stage 5 chronic kidney disease (CDK-5) should be 124–558 pg/mL, which is two to nine times the upper limit of normal (26). Treatment success was defined as serum iPTH <300 pg/mL within 7 days after treatment (15). Recurrence was defined as serum iPTH levels >558 pg/mL after successful treatment (15). Hypocalcemia and severe hypocalcemia (SH) are defined as serum Ca concentration <2.0–1.875 mmol/L and <1.875 mmol/L, respectively (normal range 2.0–2.75 mmol/L) (27).

### 2.5 Statistical analysis

Data analysis was performed using R version 4.3.1 (R Foundation, Vienna, Austria). Normally distributed continuous variables are expressed as means and standard deviations. Non-normally distributed continuous variables are expressed as medians and interquartile ranges. For categorical variables, frequencies and percentages were calculated using cross-tabulation. The groups were compared using the t-test, Wilcoxon rank-sum test, chi-squared test, or Fisher’s exact test. Differences in BMM concentration and BMD between before and 12 months after treatment were analyzed using the paired t-test or Wilcoxon signed-rank test. Between-group differences in serum iPTH, Ca, P, and ALP concentrations at each follow-up were analyzed using generalized estimating equations. All tests were two-sided, and p-values < 0.05 were considered statistically significant.

## 3 Results

### 3.1 Baseline data

Baseline demographic and clinical data are shown in **Table 1**. A total of 107 patients with SHPT (410 nodules)—48 in the MWA group and 59 in the PTX group—were enrolled in the study. The mean age was 51.2 ± 10.1 years (range, 26–75 years). There were no significant differences in sex ratio, age, dialysis history, number of nodules, maximum nodule diameter, and other markers between the two groups (p > 0.05).

**Table 1:** Baseline demographic and clinical data.

Parameters	MWA (n=48)	PTX (n=59)	P
Sex			0.838
Male (%)	30 (62.5)	38 (64.4)	
Female (%)	18 (37.5)	21 (35.6)	
Age (years)	50.25 ± 9.68	51.92 ± 10.49	0.400
Dialysis method			0.494
Hemodialysis (%)	47 (97.9)	55 (93.2)	
Peritoneal dialysis (%)	1 (2.1)	4 (6.8)	
Dialysis frequency (times/week)	3.06 ± 0.60	3.19 ± 0.97	0.421
Dialysis vintage (years)	7.24 ± 2.79	7.05 ± 2.87	0.733
Nodule number	3.92 ± 0.79	3.76 ± 0.65	0.273
Nodule maximum diameter (cm)	1.99 ± 0.40	2.10 ± 0.65	0.291
iPTH (pg/mL)	1970.88 ± 858.76	1941.13 ± 958.59	0.868
Serum calcium (mmol/L)	2.47 ± 0.25	2.50 ± 0.27	0.608
Serum phosphorus (mmol/L)	2.14 ± 0.67	2.20 ± 0.60	0.597
ALP (IU/L)	305.50 (127.25, 565.00)	203.00 (132.00, 416.00)	0.183
Creatinine (µmol/L)	931.00 (745.50, 1051.75)	949.00 (769.00, 1130.50)	0.456
Urea nitrogen (mmol/L)	24.05 (19.74, 28.11)	21.70 (15.88, 30.26)	0.250

MWA, microwave ablation; PTX, parathyroidectomy; iPTH, intact parathyroid hormone; ALP, alkaline phosphatase.

### 3.2 Treatment outcomes

In patients treated with MWA, all multiple lesions were completely ablated. The efficacy and safety of MWA and PTX are shown in **Table 2**. The success rate was marginally lower in the MWA group than in the PTX group, and the recurrence rate was slightly higher in the former; however, these differences were not significant (p > 0.05). The procedure time, intraoperative blood loss, length of hospital stay, and hospitalization costs were significantly lower in the MWA group (p < 0.001). Hypocalcemia was the most common complication after MWA and PTX, with no difference between the groups (p > 0.05). The incidence of SH was significantly higher in the PTX group (p < 0.05). There were no significant between-group differences in the incidence of hoarseness, infection/fever, and hematoma (p > 0.05).

**Table 2:** Efficacy and safety of MWA and PTX.

Parameters	MWA (n=48)	PTX (n=59)	P
Treatment success rate (%)	87.5	98.3	0.064
Recurrence rate (%)	8.3	5.1	0.777
Procedure time (min)	58.8 ± 20.4	134.4 ± 39.1	<0.001
Blood loss volume (mL)	5.0 (5.0, 5.0)	20.0 (10.0, 20.0)	<0.001
Hospital stay (days)	12.0 (8.0, 14.0)	15.0 (14.0, 20.0)	<0.001
Hospitalization cost (thousand yuan)	23.91 (21.00, 28.10)	26.03 (24.70, 28.79)	0.004
Hypocalcemia (%)	29 (60.4)	41(69.5)	0.326
Severe hypocalcemia (%)	17 (35.4)	33 (55.9)	0.034
Hoarseness (%)	7 (14.6)	3 (5.1)	0.179
Infection or fever (%)	0 (0.0)	3 (5.1)	0.319
Hematoma (%)	1 (2.1)	2 (3.4)	>0.999

MWA, microwave ablation; PTX, parathyroidectomy.

**Figure 2:**
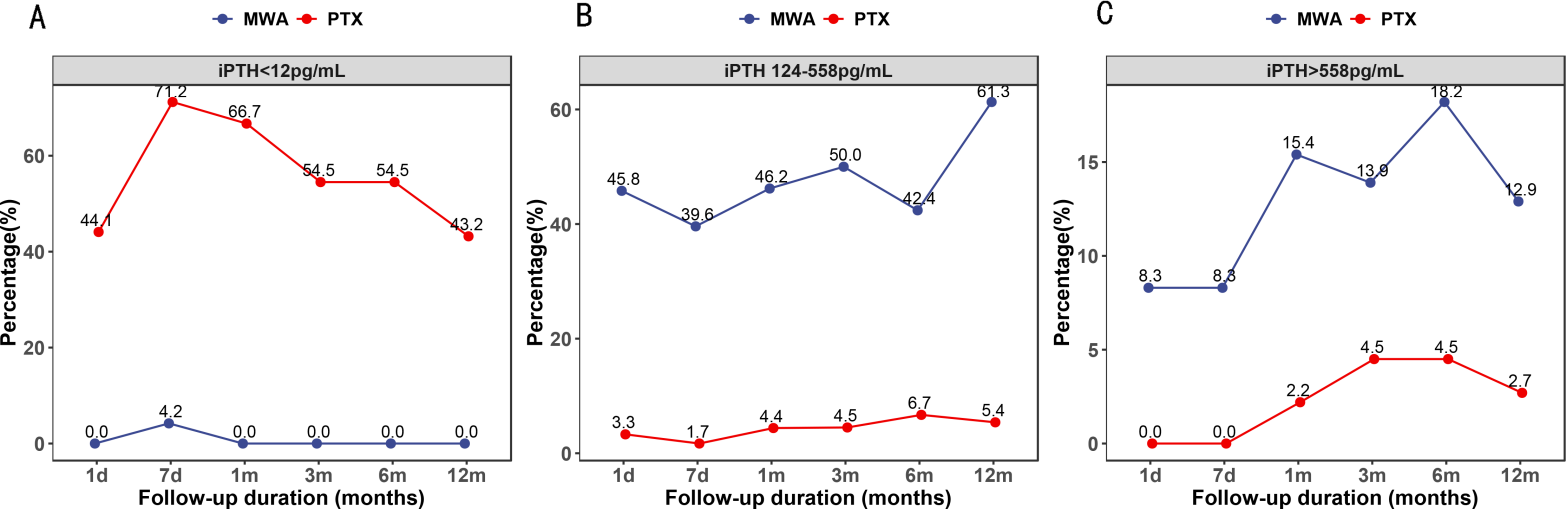
Percentage of patients whose serum intact parathyroid hormone levels were lower than 12 pg/mL **(A)**, within the target range **(B)**, and higher than 558 pg/mL **(C)**.

Post-treatment iPTH levels in the two groups are shown in **Figure 2**. The percentage of patients with iPTH <12 pg/mL was significantly lower in the MWA group at each follow-up (p < 0.05). Nevertheless, the percentage of patients whose iPTH levels were within the target range and iPTH >558 pg/mL was significantly higher in the MWA group at each follow-up (p < 0.05).

**Figure 3:**
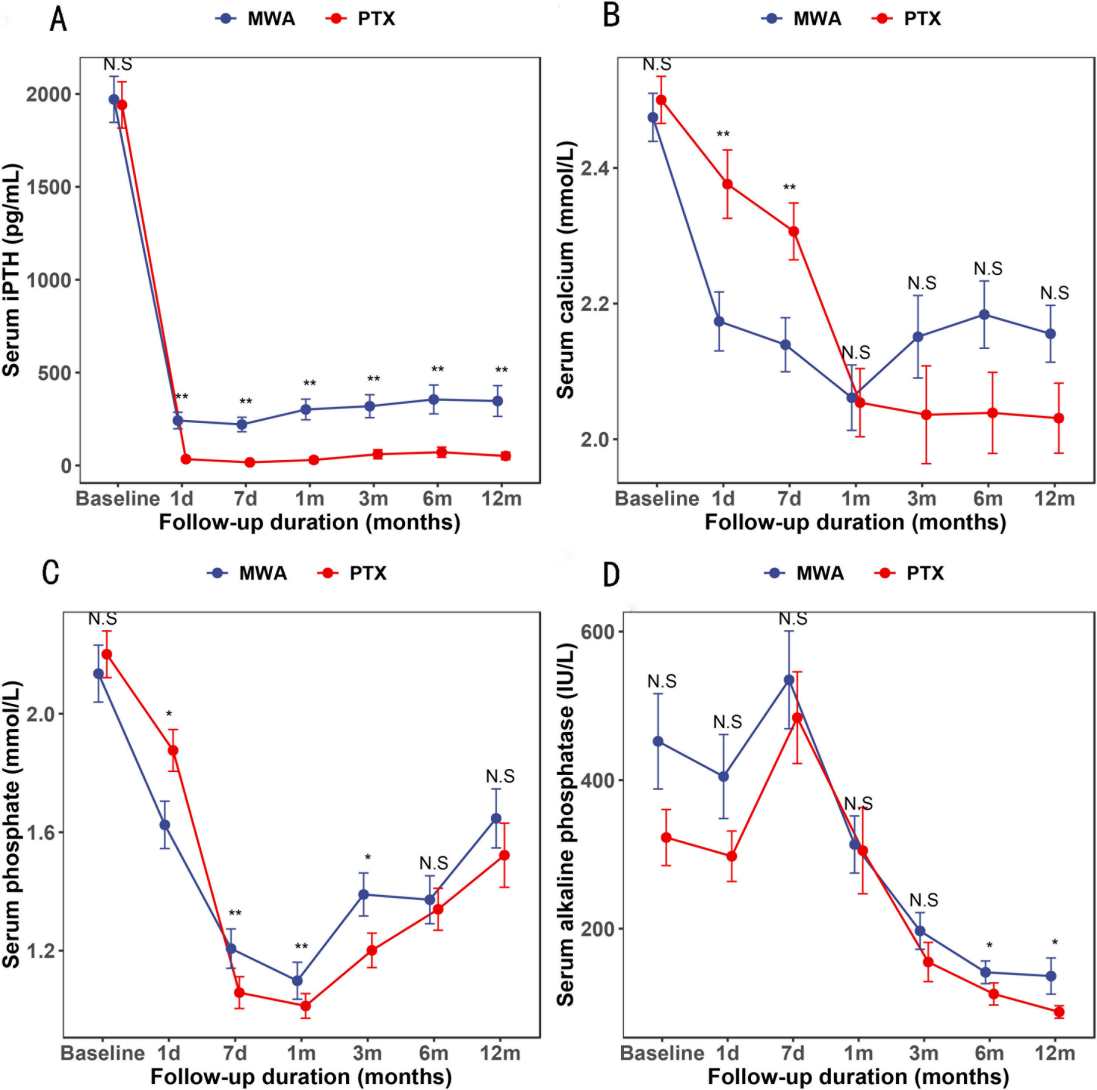
**(A–D)** Mean concentrations of intact parathyroid hormone, calcium, phosphorus, and alkaline phosphatase during the study period. *p < 0.05, ^**^p < 0.01. NS, no statistical difference.

The effects of treatments on iPTH, Ca, P, and ALP concentrations are shown in **Figure 3**. Both procedures decreased iPTH levels 1 day postoperatively, and the decrease was more pronounced after PTX (**Figure 3A**). Both therapies decreased serum Ca up to 1 month postoperatively, and the decrease was more pronounced at 1 and 7 days after MWA (**Figure 3B**). Similarly, both treatments decreased serum P up to 1 month postoperatively, and concentrations increased gradually within 12 months (**Figure 3C**). The ALP concentration in the two groups reached a peak at 7 days after treatment, after which it continued to decline. The ALP concentration was lower in the PTX group than in the MWA group at 6 and 12 months after treatment (p < 0.05) (**Figure 3D**).

### 3.3 Effect of treatments on BTMs and BMD

Changes in BTMs and BMD before and 12 months after MWA and PTX are shown in **Table 3**. There were no significant between-group differences in OC, β-CTX, 25(OH)D, and BMD before treatment (p > 0.05). However, both treatments decreased OC and β-CTX concentrations and increased 25(OH)D levels and BMD in the LS, FN, and TH at 12 months after treatment (p < 0.05), and the 25(OH)D, LS, FN, or TH showed no significant differences between the two groups (p > 0.05). Nonetheless, the effect of PTX on OC and β-CTX levels was more pronounced.

**Table 3:** Bone turnover markers and bone mineral density pre- and post-treatment.

Parameters	Baseline	12 months	P_1_
OC (ng/mL)			
MWA	226.17 ± 43.83	131.95 ± 50.54	<0.001
PTX	222.71 ± 51.44	79.28 ± 39.00	<0.001
P_2_	0.715	<0.001	
β-CTX (ng/mL)			
MWA	5.38 ± 1.05	1.75 ± 0.90	<0.001
PTX	5.33 ± 1.27	0.94 ± 0.53	<0.001
P_2_	0.820	<0.001	
25(OH)D (nmol/L)			
MWA	62.65 (50.85, 78.85)	78.70 (68.48, 87.27)	0.011
PTX	61.65 (48.10, 79.55)	81.80 (70.15, 94.45)	<0.001
P_2_	0.977	0.306	
Lumbar spine			
MWA	-1.70 (-2.45, -0.98)	-0.75 (-1.75, -0.12)	<0.001
PTX	-2.10 (-2.80, -0.90)	-1.10 (-1.65, -0.05)	<0.001
P_2_	0.418	0.986	
Femoral neck			
MWA	-2.00 ± 0.86	-1.18 ± 0.87	<0.001
PTX	-2.04 ± 0.99	-1.03 ± 0.98	<0.001
P_2_	0.839	0.536	
Total hip			
MWA	-1.55 ± 0.92	-0.98 ± 0.81	<0.001
PTX	-1.68 ± 0.95	-0.93 ± 0.92	<0.001
P_2_	0.489	0.830	

MWA, microwave ablation; PTX, parathyroidectomy; OC, osteocalcin; β-CTX, beta C-terminal cross-linked telopeptide of type I collagen; 25(OH)D, 25-hydroxy vitamin D; P_1_: comparison between before and after surgery; P_2_: comparison between MWA and PTX.

## 4 Discussion

The results showed that MWA and PTX decreased serum iPTH, Ca, P, ALP, OC, and β-CTX and increased serum 25(OH)D levels and BMD in the LS, FN, and TH in patients with SHPT. Both therapies corrected bone metabolism disorders and improved bone density.

The KDIGO recommends that iPTH levels in patients with CDK-5 should not be over-suppressed (26). Sustained low iPTH concentrations may be related to reduced bone formation and an increased incidence of dynamic bone disease, bone pain, and fractures; thus, maintaining ideal iPTH levels is essential for bone turnover (28, 29). Although MWA and PTX reduced iPTH levels, the effect of PTX was more pronounced. Moreover, the percentage of patients whose iPTH concentration was within the target range was higher in the MWA group, and the percentage of patients with iPTH <12 pg/mL was lower, consistent with previous studies (12, 14). This difference may be related to two factors. First, PTX is performed under direct vision; thus, removing parathyroid tissue is easier, whereas thermal ablation can only ablate the hyperplasia of parathyroid tissue identified on ultrasound. Second, although post-ablation CEUS can reveal the active portion of nodules, there may still be residual marginal lesions that cannot be detected by ultrasound.

ALP and OC are markers of osteoblast activity and bone formation (30, 31) while CTX is a marker of osteoclast activity and bone resorption (22, 32). These markers combined reflect bone metabolism status. We found that preoperative ALP, OC, and β-CTX levels were significantly increased in our patients, indicating active bone formation and resorption. Consistent with this finding, Woitge et al. showed that OC and CTX were elevated in patients with renal insufficiency (33). Although MWA and PTX reduced ALP, OC, and β-CTX, the effect of PTX was more pronounced, indicating that both therapies can stimulate bone formation and resorption and maintain bone homeostasis. Among these indicators, ALP showed a brief and significant increase within 7 days after treatment, after which it continued to decline. Similarly, other studies found that PTX caused a short-term increase in ALP in patients with SHPT (22, 23). Moreover, Yajima et al. observed that a rapid decline in iPTH can inhibit bone resorption and cause a significant transient increase in bone formation, as observed using bone biopsy 1 week after PTX (34). This result may explain the transient elevation in ALP levels within 7 days after MWA and PTX.

High bone turnover in SHPT can decrease BMD and is the leading cause of metabolic bone diseases in patients with renal insufficiency (35). Hyperparathyroidism is usually represented as the bone density is lower in the cortical than the trabecular bone (36). We found that baseline BMD was low in most of our patients, with lower BMD in the LS and FN compared to that in TH, and PTX increased BMD, especially in the LS and FN, consistent with the literature (24, 25, 37), suggesting that sites with lower BMD may benefit more from treatment in these patients.

MWA also increased BMD, although the degree of increase was slightly lower than that of PTX. Similarly, Wu et al. found that MWA increased BMD in the LS and FN within 2 years after surgery in patients with primary hyperparathyroidism (pHPT) (38). While the mechanism underlying this increase in BMD remains unclear, it may be related to improvements in biochemical characteristics after PTX (39, 40). Therefore, MWA is also a therapy that can promote bone recovery. However, Nomura et al. found that BMD in patients with pHPT increased significantly and continuously within 6 years after PTX (41). Therefore, we predicted that BMD may also continue to increase over a long period after surgery in patients with SHPT, although longer follow-up is needed to verify the difference between MWA and PTX in improving BMD.

Our data showed that hypocalcemia was the most common complication following PTX and MWA, with no significant differences in incidence rate between the two groups, although PTX was associated with a higher incidence of SH. A possible explanation is that iPTH decreases more rapidly and strongly after PTX than after MWA, leading to a faster transfer of large amounts of serum Ca to previously decalcified bones, resulting in a significant decrease in serum Ca concentrations. Wei et al. found that the number of removed parathyroid glands was an independent risk factor for postoperative SH in patients with SHPT (27). MWA can ablate parathyroid tissue that can be shown on ultrasound, but there may still be a small number of residual lesions. Although the PTX group in this study underwent AT, the iPTH concentration was still significantly lower than that of MWA, which may be related to the non-survival of transplanted parathyroid tissue. A meta-analysis found that the incidence of hypocalcemia was similar between tPTX+AT and sPTX; nonetheless, the former was associated with an increased incidence of severe symptomatic hypocalcemia (42). The incidence of other complications, such as hoarseness, infection/fever, and hematoma, was similar between the two groups, demonstrating that MWA is safe for treating SHPT.

This study has limitations. First, the design was retrospective and had a limited sample size. Second, the follow-up time was short; thus, we were unable to investigate the long-term efficacy and recurrence rates. To address these limitations, future studies with increased sample sizes and longer follow-up times are needed to explore the long-term efficacy of these procedures. In addition, BTM and BMD data were only available before and 12 months after treatment in this study, and the bone density changes in the forearm are not mentioned, although cortical bone loss occurs more usually in SPTH and should be perfected in future studies.

In conclusion, MWA and PTX are effective and safe methods for treating SHPT, and both improve bone metabolism markers and BMD. MWA is a minimally invasive percutaneous approach with great potential for treating patients with SHPT who cannot tolerate PTX. Based on the findings of this study, it is necessary to consider the individual differences of patients in the treatment of SPTH and select the most appropriate option.

## Data Availability

The raw data supporting the conclusions of this article will be made available by the authors, without undue reservation. Requests to access these datasets should be directed to Jiantang Zhang, 450253348@qq.com.
